# Bcl11b/Ctip2 in Skin, Tooth, and Craniofacial System

**DOI:** 10.3389/fcell.2020.581674

**Published:** 2020-12-10

**Authors:** Marie-Thérèse Daher, Pedro Bausero, Onnik Agbulut, Zhenlin Li, Ara Parlakian

**Affiliations:** Biological Adaptation and Ageing, Inserm ERL U1164, UMR CNRS 8256, Institut de Biologie Paris-Seine, Sorbonne Université, Paris, France

**Keywords:** BCl11B, skin, lipid metabolism, tooth morphology, craniosynostosis, SNP, cardiovascular system

## Abstract

Ctip2/Bcl11b is a zinc finger transcription factor with dual action (repression/activation) that couples epigenetic regulation to gene transcription during the development of various tissues. It is involved in a variety of physiological responses under healthy and pathological conditions. Its role and mechanisms of action are best characterized in the immune and nervous systems. Furthermore, its implication in the development and homeostasis of other various tissues has also been reported. In the present review, we describe its role in skin development, adipogenesis, tooth formation and cranial suture ossification. Experimental data from several studies demonstrate the involvement of Bcl11b in the control of the balance between cell proliferation and differentiation during organ formation and repair, and more specifically in the context of stem cell self-renewal and fate determination. The impact of mutations in the coding sequences of Bcl11b on the development of diseases such as craniosynostosis is also presented. Finally, we discuss genome-wide association studies that suggest a potential influence of single nucleotide polymorphisms found in the 3’ regulatory region of Bcl11b on the homeostasis of the cardiovascular system.

## Introduction

Since the discovery of the first mammalian transcription factor Sp1 (a zinc finger protein) and its corresponding DNA binding sequence (GC boxes) five decades ago (Dynan and Tjian, 1983), many other transcription factors with remarkable and complex features have been identified and have led to our current understanding of gene regulation/expression. For example, treatment of fibroblasts with the DNA demethylating agent 5-Azacytidine resulted in a change in cell fate mediated by the transcription factor MyoD, a master regulatory gene for myogenic differentiation (Lassar et al., 1986). In 2006, the combination of 4 transcription factors induced the dedifferentiation of terminally differentiated cells into induced pluripotent stem cells (iPS) (Takahashi and Yamanaka, 2006). These studies and many others led to the notion that transcription factors play a crucial role in coupling actors of epigenetic regulation to gene expression and consequently cell fate determination and tissue homeostasis. The identification of chromatin remodeling complexes such as the NuRD complex and the SWI/SNF complex and their ability to interact with various transcription factors reinforced this concept (Kwon et al., 1994; Ryan et al., 1998; Xue et al., 1998; Tyler et al., 1999). One such transcription factor is CTIP2 (COUP-TF-interacting protein 2), also known as Bcl11b (B-cell lymphoma/leukemia 11B) or Rit1 (radiation-induced tumor suppressor gene 1), on which we will be focusing in the present review. Following a brief overview of its discovery, binding partners and molecular mechanisms of action, we focus on the physiological role of Bcl11b in murine models and human pathologies when data is available. More specifically, we describe its role in skin homeostasis, adipogenesis, tooth formation, and craniofacial skeleton growth. We discuss also data from recent genome-wide association studies (GWAS) that suggest to some extent a potential influence for this gene in modulating the occurrence of cardiovascular disease. The function of Bcl11b in T cell biology and the brain is largely covered by numerous reviews (Liu et al., 2010; Avram and Califano, 2014; Lennon et al., 2017; Rothenberg, 2019; Simon et al., 2020) and is presented only briefly in the present review.

## General Features of CTIP2/BCL11B

### An Overview of Its Role in the Immune and Nervous Systems

Murine CTIP2, along with CTIP1, was first identified as a zinc finger protein implicated in transcriptional repression by interacting with the COUP-TF orphan nuclear receptors protein family (Avram et al., 2000). It was “rediscovered” independently as a tumor suppressor gene based on allelic loss mapping on chromosome 12 in γ-ray induced mouse thymic lymphomas (Wakabayashi et al., 2003a). Its human ortholog (Rit1/Bcl11b) was described in human gene databases and mapped to chromosome 14 (14q32.1) (Satterwhite et al., 2001). It was later reported that these chromosomal loci undergo recurrent cryptic chromosomal rearrangements t(5;14). These translocations induce ectopic expression of TLX3 or NKX2.5 genes (Ch. 5) in T cells by the juxtaposition of regulatory sequences found at the 3’ region of the Bcl11b gene in T-ALL (acute lymphoblastic leukemia) patients (Bernard et al., 2001; MacLeod et al., 2003; Nagel et al., 2003, 2007; Su et al., 2006; Van Vlierberghe et al., 2008; De Keersmaecker et al., 2010). Additional chromosomal rearrangements were also reported in adult T-cell leukemia/lymphoma (ATLL) t(2;14)(q34;q32) and acute myeloid leukemia (AML) t(2;14)(q22.3;q32.2) leading respectively to the formation of a HELIOS-BCL11B (Fujimoto et al., 2012) and a ZEB2-BCL11B fusion protein (Torkildsen et al., 2015; Padella et al., 2019). Subsequent studies on Bcl11b-deficient mice revealed its primary role in T-cell biology (Wakabayashi et al., 2003b; Inoue et al., 2006; Albu et al., 2007; Ikawa et al., 2010; Kastner et al., 2010; Li et al., 2010a,b; Hirose et al., 2015; Hosokawa et al., 2018, 2020; Hu et al., 2018). In mouse embryo, Bcl11b mRNA was first detected in the nasal cavity epithelium and in the outer layers of the cerebral cortex at E10.5. Bcl11b was also found to be expressed at high levels in the thymus at E14.5 (Leid et al., 2004). Expression of Bcl11b in thymocytes is initiated at the DN2 stage of T cell development, activated by Notch signaling and transcription factors such as TCF-1, GATA3 and Runx1 (Tydell et al., 2007; Weber et al., 2011; Li et al., 2013; Kueh et al., 2016; Longabaugh et al., 2017; Garcia-Perez et al., 2020). Following an initial report on its role in the differentiation and survival of αβ T lymphocytes (Wakabayashi et al., 2003b), a study by the group of D. Avram showed that Bcl11b plays a critical role for positive selection of both CD4 and CD8 lineages as well as its requirement for the survival of double-positive thymocytes (Albu et al., 2007). Indeed, in the absence of Bcl11b, double positive thymocytes show increased susceptibility for apoptosis and express higher levels of cleaved caspase-3. It has been further demonstrated that Bcl11b-deficient thymocytes are blocked in the multipotent double-negative DN2 (CD4^–^ CD8^–^) stage of T-cell specification and fail to progress into the DN3 (CD44^–^ CD25^+^) stage (Ikawa et al., 2010; Li et al., 2010a,b). In addition, these cells retain their ability to differentiate into other cell fates such as natural killer-like cells and maintain the expression of stem cell characteristic genes. Its role in the formation of specific T-cell subsets at different stages of T-cell development and maturation was further investigated. Bcl11b was shown to be required for the development of γδ T-cells (Shibata et al., 2014; Hatano et al., 2017; Dolens et al., 2020), iNKT cells and T-regulatory cells (Albu et al., 2011; Vanvalkenburgh et al., 2011; Uddin et al., 2016; Drashansky et al., 2019; Hasan et al., 2019). Additionally, Bcl11b is involved in the differentiation of CD4 + CD8- helper T cells and CD4-CD8 + cytotoxic T cells through the regulation of key transcription factors ThPOK and Runx3 (Kojo et al., 2017, 2018). Furthermore, the suppression of Bcl11b protein from mature T cells leads to the occurrence of multiple defects, including the derepression of the Th2 program in Th17 cells (Califano et al., 2014), the failure of Th2 cells to differentiate during asthma and antihelminth responses (Lorentsen et al., 2018), and the failure of efficient CD8 + T cell expansion in response to infection with Listeria monocytogenes or Vaccinia virus (Zhang et al., 2010; Abboud et al., 2016). Bcl11b, is also expressed in group 2 innate lymphoid cells (ILC2s), where it maintains mature ILC2 program and contributes to the repression of ILC3-specific genes in ILC2 cells (Cismasiu et al., 2006; Califano et al., 2015; Walker et al., 2015, 2019; Yu et al., 2015, 2016; Xu et al., 2019). It has been shown also that hematopoietic progenitors fail to generate ILC2 cells upon Bcl11b inactivation (Yu et al., 2015, 2016).

The function of Bcl11b in neuronal networks and the nervous system is also largely studied. Bcl11b is specifically expressed in corticospinal motor neurons (CSMN) mainly localized in cortical layer V (Arlotta et al., 2005). Based on the phenotype of Bcl11b-deficient mice, it was shown that Bcl11b is not necessary for the early specification of cortical precursors but is required for the establishment of cortical connections to the spinal cord. Indeed, Bcl11b-deficient CSMN axons show defects in fasciculation, outgrowth, and path finding processes that are crucial for corticospinal connections. Bcl11b was also required for the differentiation of striatal medium spiny motoneurons and its potential role in Huntington disease-related transcriptional deregulation was suggested (Arlotta et al., 2008; Fjodorova et al., 2019). Additional studies showed the implication of Bcl11b in spatial working memory by regulating neurogenesis in the hippocampus (Simon et al., 2012, 2016; De Bruyckere et al., 2018). Conditional deletion of Bcl11b in the adult mouse hippocampus leads to a rapid loss of excitatory synapses and the deregulation of genes associated with synaptic transmission (De Bruyckere et al., 2018). Bcl11b is also involved in the negative regulation of glial progenitor cell differentiation (Wang et al., 2018).

### Mechanisms of Action, Partner Proteins, and Target Genes

As previously mentioned, initial reports on Bcl11b classified this transcription factor in the category of “transcriptional repressors”. It was initially reported that Bcl11b-mediated transcriptional repression was enhanced by recruitment of SIRT1 (Senawong et al., 2003). Transcriptional silencing of HIV-1 is one of many examples for Bcl11b-mediated transcriptional regulation. It has also been reported that ectopic expression of Bcl11b inhibits Sp1- and COUP-TF-mediated activation of HIV-1 gene transcription and related viral replication in microglial cells (Marban et al., 2005). Furthermore, HIV1-latent patients showed higher levels of BCL11b and other chromatin modifiers (HP1α, MeCP2, and HDAC1) involved in silencing in the brain (Desplats et al., 2013). Through the sequential recruitment of histone deacetylases (HDACs 1 and 2), SUV39H1 methyltransferase and HP1 on chromatin, Bcl11b contributes to the formation of a heterochromatic environment at the HIV-1 promoter and to the generation of H3K9me3 epigenetic mark (Marban et al., 2007; for review see Wallet et al., 2019). Therefore, Bcl11b allows for the virus to be maintained in cellular reservoirs. This mode of silencing was also described for cellular targets of Bcl11b such as the cell cycle inhibitor gene p21 (Cherrier et al., 2009). In T lymphocytes, by binding directly to the HIV-1 LTR, Bcl11b represses transcription from the LTR via its interaction with components of the major transcriptional corepressor complex NuRD (nucleosome remodeling and deacetylation), such as MTA1 and MTA2 (Cismasiu et al., 2005, 2008). The NuRD complex-mediated transcriptional repression by BCL11b also impacts p57Kip2, SPRY1 gene expression (Topark-Ngarm et al., 2006; Wiles et al., 2013).

An additional study showed that Bcl11b, through its interaction with the pTEFb regulatory complex (CyclinT1/CDK9) containing the HEXIM1, 7SK snRNA and HMGA1, participates in the repression of RNA polymerase II elongation activity (Cherrier et al., 2013; Eilebrecht et al., 2014). The study shows that Bcl11b and HMGA1 synergistically repress the HIV-1 promoter activity (Eilebrecht et al., 2014). Post-translational modification is another characteristic feature of Bcl11b. Indeed, Bcl11b undergoes MAP kinase-dependent multisite phosphorylation coupled with SUMOylation after the treatment of native thymocytes with phorbol 12,13-dibutyrate/calcium ionophore A23187 (Zhang et al., 2012b; Vogel et al., 2014). The SUMOylation was shown to modify Bcl11b activity by allowing the recruitment of the transcriptional co-activator p300 (a histone acetyltransferase) and activate target gene transcription such as Id2 (Zhang et al., 2012b). During TCR activation of human CD4 T cells, phosphorylation of BCL11b by protein kinase C decreases its interaction with components of the NuRD complex and allows the recruitment of p300, switching BCL11b from a transcriptional repressor to an activator (Dubuissez et al., 2016). Finally, the overexpression of KLF4 induces the SUMOylation of BCL11b, this time leading to its degradation and decreased expression of T cell-associated genes such as NOTCH1, GATA3 and TCF7 in Jurkat cells (Li et al., 2015).

In addition to the above-mentioned Bcl11b target genes (Id2, p21, p57kip2 and the HIV-1 promoter), several other targets were uncovered in various tissues using ChIP (chromatin immunoprecipitation) experiments (some of these targets are mentioned in the paragraphs corresponding to the different tissues). Bcl11b has initially been shown to bind a core GC-rich motif, GGCCGG (Avram et al., 2002; Cismasiu et al., 2006; Topark-Ngarm et al., 2006). A genome-wide expression profile coupled with a ChIP-sequencing approach identified 247 direct targets (149 down-regulation and 98 up-regulation) of Bcl11b potential target genes in striatal cells of the nervous system *in vitro*. Many of these target genes are implicated in brain-derived neurotrophic factor (BDNF) signaling, whose activity is tightly linked to neurodegenerative syndromes such as Huntington’s, Alzheimer’s and Parkinson’s diseases (Tang et al., 2011). This study led to the identification of novel Bcl11b DNA binding sites (ACCACA, TGCTTG, AGTGCT, AG[AT]GTG and GGATCA) that, to some extent, share homology with consensus binding motifs for AML1, MAFB, and HNF4 transcription factors. Studies on the role of Bcl11b in Treg cells of both humans and mice identified more than 500 targets for Bcl11b. Interestingly, the top 2 enriched motifs for Bcl11b binding were Ets and Runx binding motifs (Drashansky et al., 2019; Hasan et al., 2019). Similarly, more than 900 and 94 Bcl11b targets were observed in pro-T cells and ILC2 cells, respectively (Hosokawa et al., 2020). In pro-T cells, the top 2 enriched motifs once again were Runx and Ets while bZIP and Ets motifs were found in ILC2 cells. In this context, distinct Bcl11b protein partners and different sets of genes controlled by Bcl11b were observed in pro-T and ILC2 cells, indicating that the same transcription factors can regulate substantially different target genes in the two different cells (Hosokawa et al., 2018, 2020). The results obtained in Treg cells indicated that Bcl11b, in association with Foxp3, is primarily responsible in establishing a Treg-specific gene activation program (Drashansky et al., 2019). Removal of Bcl11b in Treg cells resulted in multiorgan fatal autoimmunity and lymphadenopathy (Drashansky et al., 2019; Hasan et al., 2019). Genome-wide binding analysis of Bcl11b revealed that many Bcl11b binding sites were found to overlap with Foxp3 binding sites, suggesting that Bcl11b and Foxp3 are interdependent and cooperate to control the Treg program. In addition, ATAC-seq experiments show that Bcl11b maintains chromatin accessibility at Treg signature genes in Treg cells. Interestingly, Bcl11b showed a similar binding profile in mouse naive CD4 + T cells. However, in the absence of Foxp3, this binding profile was correlated with a repressive state of the corresponding genes.

Taken together, these data indicate that Bcl11b functions as a transcriptional repressor when associated with multiple partners implicated in chromatin remodeling such as Sirtuins, HDACs, the NurD complex and the SWI/SNF complex (Avram et al., 2000, 2002; Senawong et al., 2003; Cismasiu et al., 2005; Kadoch et al., 2013; Sokpor et al., 2017). Bcl11b also acts as a transcriptional activator when associated with the p300 histone acetyl transferase (Cismasiu et al., 2006; Zhang et al., 2012b). Expression of Id2 illustrates the chameleon-like behavior of Bcl11b; it participates in the inhibition of Id2 expression in the T-cell precursor (Hosokawa et al., 2018) and switches to being an activator for Id2 expression upon its SUMOylation in activated T cells (Zhang et al., 2012b).

## Physiological Role of BCL11B During Organ Formation

### Bcl11b Maintains Skin Homeostasis

The role of Bcl11b in skin homeostasis and disease was evidenced through a series of studies using constitutive or tissue-specific Bcl11b-deficient mice models as well as the descriptive analysis of its expression under pathological conditions in humans. Its implication in skin cancer-related pathways was also investigated. Bcl11b expression in murine skin was first reported by Golonzhka et al. (2007, 2009a). Its expression is detected during murine embryonic development as early as E10.5 in the ectoderm. Later in development, it becomes restricted to the proliferating basal cell layer of the epidermis and in some cells of the suprabasal layer. A similar distribution, but lower expression, of Bcl11b was observed in adult murine skin. The same pattern of expression was also reported for human skin (Ganguli-Indra et al., 2009a).

**FIGURE 1:**
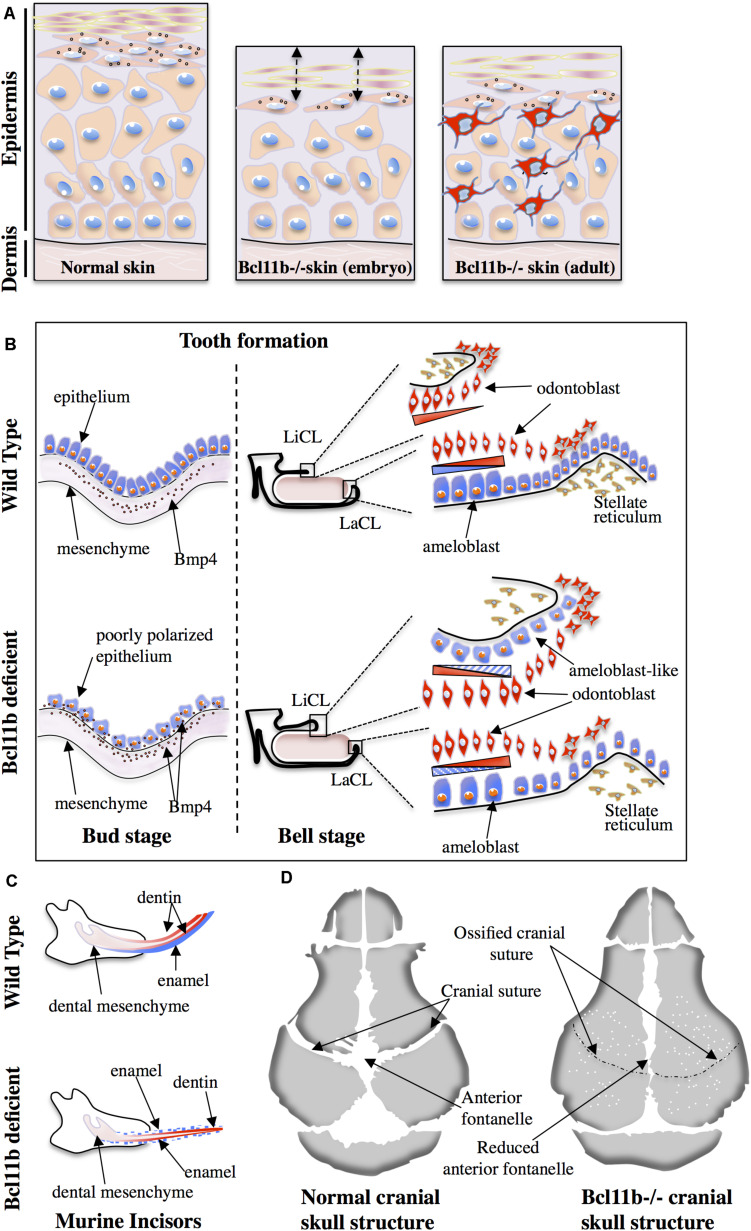
Role of Bcl11b in skin **(A)**, tooth **(B,C)** and cranial suture **(D)** ossification. **(A)** The epithelial thickness and number of basal keratinocytes are reduced in Bcl11b-deficient embryonic murine skin (middle panel) compared to wild-type skin (left panel). Compromised epithelial barrier is also a characteristic feature of Bcl11b-deficient embryonic skin (double-headed arrows). Adult Bcl11b deficient skin (right panel) shows an inflammatory phenotype with the infiltration of immune/inflammatory cells (in red). **(B)** Poorly polarized epithelium and maintained presence of Bmp4 expression in epithelial cells are the two main features of Bcl11b-deficient mice at the bud stage (left panel). At the bell stage (right panel), contrary to the wild type, Bcl11b-deficient mice show a small labial cervical loop (LaCL) and bigger lingual cervical loop (LiCL). The presence of ameloblast-like cells (in blue) on the lingual side leads to the loss of asymmetry during incisor formation. **(C)** Asymmetric formation of incisors in wild-type mice is compromised in Bcl11b-deficient mice. Note the presence of enamel (blue) on both labial and lingual sides of incisors in mutant mice. **(D)** Accelerated ossification of cranial sutures and reduced anterior fontanelle in Bcl11b-deficient mice lead to craniosynostosis. White dots indicate bone fragility in mutant mice.

The major finding of the murine studies was the implication of Bcl11b in the establishment of the epidermal permeability barrier (Figure 1A) through both cell autonomous (regulation of keratinocyte proliferation and differentiation) and non-cell autonomous (regulation of paracrine growth factors) mechanisms based on observations in either total knock-out (KO) or keratinocyte-specific KO of Bcl11b fetuses at E17.5 (Golonzhka et al., 2009a). The results observed in embryos from E14.5 to E18.5 as well as in primary keratinocytes isolated from newborn mice indicate that the regulation of epidermal proliferation/differentiation relies on Bcl11b-dependent expression of EGFR and Notch1 as well as its post-translational modifications (phosphorylation and SUMOylation) (Zhang et al., 2012a). The authors also reported the development of an atopic dermatitis (AD)-like skin inflammation phenotype in adult keratinocyte-specific KO of Bcl11b mice (Wang et al., 2012). In this study, the authors uncovered a new role of Bcl11b in repressing the expression of thymic stromal lymphopoietin (TSLP); a key protein potentially involved in the pro-inflammatory response of AD patients. The aberrant expression of Bcl11b in the skin of human AD patients suggests a critical role of this protein in the progression of this pathology (Ganguli-Indra et al., 2009a). Finally, defects in lipid distribution and dysregulated expression of lipid-processing enzymes such as Smpd3, Dgat2, Elovl4 and eLox3 were also observed in the skin of Bcl11b mutant fetuses (Golonzhka et al., 2009a).

In a separate study, the same authors determined the involvement of two new factors in addition to the previously mentioned factors EGFR and Notch1; Nfatc1 and Lhx2. These also play a role in the maintenance of hair follicle stem cells (Bhattacharya et al., 2015). The absence of Bcl11b in skin epithelial cells leads to enhanced Nfatc1 and Lhx2-dependent activation of bulge stem cells and subsequent rapid depletion. In line with these findings, the deletion of Bcl11b in the skin epidermis results in delayed wound healing and aberrant expression of hair follicle stem cell markers (Liang et al., 2012). Several other transcription factors such as p63 and MAF:MAFB play a crucial role in skin formation and homeostasis.

Zhang et al. (2012a) have showed that Bcl11b expression is regulated by calcium signaling pathways in epidermal keratinocytes. The treatment of differentiating keratinocytes with high levels of calcium lead to the decrease of Bcl11b protein levels. This response resembles that of the transcription factor ΔNp63, an isoform of P63, which is also downregulated upon treatment of keratinocytes with high calcium (Vivo et al., 2009). The p63 protein is a member of the p53 family of transcription factors. It is a master regulator of epidermal commitment and differentiation. Mice lacking p63 exhibit major defects in their limb, craniofacial and epithelial development (Mills et al., 1999; Yang et al., 1999). It has been demonstrated that p63 is essential for the expression of a subset of epidermal genes as well as for the repression of non-epidermal gene expression through temporal- and spatial-specific active enhancers (Shalom-Feuerstein et al., 2011; Bao et al., 2015; Kouwenhoven et al., 2015). The p63 protein is involved in the epigenetic regulation of epidermal keratinocytes through its participation in the recruitment of HDAC1/2 (LeBoeuf et al., 2010). Furthermore, the p63 protein exerts epigenetic control on epidermal keratinocytes through the regulation of several chromatin-remodeling factors, such as Satb1, Brg1 and Cbx4, that are direct p63 targets; their expression is markedly decreased in the p63-deficient epidermis (Fessing et al., 2011; Mardaryev et al., 2014, 2016). In addition to the epidermal master transcription factor p63, transcription factors such as MAF and MAFB (MAF:MAFB) have been recently reported as the key regulators in epidermal differentiation (Lopez-Pajares et al., 2015). MAF:MAFB are components of the AP-1 superfamily responsible for regulating the expression of many transcription factors, such as CEBPA, HOPX, UHRF1, ETS1, ZNF750, GRHL3, and KLF4, that are known to be involved in epidermal homeostasis. They established that lncRNA ANCR and TINCR act as an upstream repressor and activator of MAF:MAFB expression, respectively. They found that the p53/p63 motif is highly enriched adjacent to MAF:MAFB-binding genomic regions, such as the promoters of KLF4, ZNF750, and GRHL3, suggesting that MAF:MAFB cooperates with p63 to induce these transcription factors as well as a subset of p63 targets during epidermal differentiation (Lopez-Pajares et al., 2015). An interaction between Bcl11B and p53 has been reported for HDM2 expression in mouse thymocytes (Obata et al., 2012). It will be very interesting to investigate the potential interaction between Bcl11b and p63 or MAF:MAFB, and to define the position of Bcl11b in the regulatory network of epidermal keratinocytes.

Several human genetic studies suggest that Bcl11b could act as a tumor suppressor gene in T-cells. Chromosomal rearrangements, such as translocation and deletions leading to the loss of function of Bcl11b, were reported in 10% of studied T-ALL cases (Nagel et al., 2003; Przybylski et al., 2005; Van Vlierberghe et al., 2008; Gutierrez et al., 2011). Few reports of human studies are available regarding a potential role of Bcl11b in skin cancer. In one such study, upregulated expression of Bcl11b in head and neck squamous cell carcinoma was reported (Ganguli-Indra et al., 2009b). They found that increased Bcl11b expression was associated with poorly differentiated tumors and was colocalized with the cancer stem cell marker BMI1. In mice, the ablation of Bcl11b in the adult epidermis led to an increased risk of tumor formation upon treatment with acute doses of TPA (12-O-tetradecanoyl phorbol-13-acetate) or UVB (Bhattacharya et al., 2017). Mycosis fungoides (MF) is an additional skin cancer type where Bcl11b expression was analyzed, which accounts for almost 70% of cutaneous T-cell lymphoma (CTCL) (Gu et al., 2013). The major finding of the study demonstrated that Bcl11b is overexpressed in all stages of MF as compared to benign inflammatory dermatosis and its expression was downregulated upon interferon treatment leading to increased cancer cell apoptosis. In a similar context, HDAC inhibitors were tested in various CTCL cell lines (Fu et al., 2017). Cell lines that express high levels of Bcl11b showed a higher sensitivity for apoptosis upon HDACi treatment, suggesting a positive correlation between these two parameters. Taken together, these studies establish a critical role for Bcl11b in the maintenance of skin homeostasis and stem cell biology through the regulation of key transcription and secreted factors. Altered expression of this gene in some inflammatory skin disorders, wound healing and in tumors suggests its role in these pathologies.

### Bcl11b Involvement in Lipid Metabolism and Adipogenesis

The presence of high amounts of lipid at the outer layer of the skin (stratum corneum) is crucial for the establishment and function of the skin barrier. The stratum corneum contains various types of lipids, the most abundant being ceramides in addition to sphingomyelins, cholesterol and fatty acids. In line with its role in the establishment of the epidermal permeability barrier, Bcl11b was shown to control sphingolipid metabolism through direct or indirect regulation of genes that encode key enzymes involved in sphingolipid synthesis (Bollag, 2013; Wang et al., 2013). Indeed, embryonic epidermal lipid composition was modified upon deletion of Bcl11b. These modifications were associated with an alteration in the expression profile of key enzymes implicated in lipid metabolism. Through a combination of q-PCR, Western blot and ChIP experiments, the study showed that three crucial lipid metabolizing enzymes, Lass2, Gba2 and eLox3, were direct Bcl11b targets (Wang et al., 2013).

The role of Bcl11b in adipocyte formation and maintenance was “accidentally” uncovered. Inoue et al. described the role of Bcl11b as a regulator of adipogenesis during embryonic development (Inoue et al., 2016). Using a series of *in vivo* and *in vitro* approaches, they initially observed lower amounts of subcutaneous white adipose tissue (WAT) in Bcl11b-deficient embryos. *In vitro*, shRNA-mediated Bcl11b downregulation in preadipocytes led to impaired adipocyte formation. At the molecular level, the expression of key transcription factors (*PPAR*γ*2, C/EBP*α) as well as differentiation markers (*perilipin*, *aP2*) implicated in adipogenesis were downregulated in Bcl11b-deficient cells. Finally, the authors suggest an interesting role for Bcl11b in the inhibition of the Wnt/β-catenin pathway, which is required for adipogenic cell fate determination (Christodoulides et al., 2009). Given the lethal phenotype of Bcl11b-deficient mice (*Bcl11b*−/− mice die within 24 h after birth), the same authors analyzed the role of Bcl11b in adipocytes of *Bcl11b* ± heterozygous mice (Inoue et al., 2017). Using a C57BL/6 × BALB/c mix background to avoid dentition-related food intake problems, the study reported a slight decrease in the weight of *Bcl11b* ± heterozygous mice as compared to wild type. The weight difference became increasingly evident when mice were challenged with a high fat diet (HFD). Indeed, *Bcl11b* ± heterozygous mice remained leaner than control mice when fed with HFD and were characterized by smaller adipocytes in subcutaneous and epididymal WAT as well as smaller lipid droplets in the liver. No marked differences were found in the expression of most adipocyte differentiation markers. However, energy consumption increased in heterozygous mice both under standard and HFD feeding protocols. The molecular mechanisms involved in increased energy consumption remain to be elucidated. The discovery of new Bcl11b-dependent pathways that regulate WAT mass and energy consumption could be of potential interest in the field of metabolic diseases and obesity-related research.

### Influence of Bcl11b in Tooth Formation and Growth

During embryogenesis, tooth development in mice is initiated by the thickening of the oral ectoderm at 10.5-day-old embryos (E10.5), and is characterized by the coordinated interaction between the ectoderm and the underlying mesoderm during the bud stage (E12.5). The folding of the dental ectoderm occurs during the cap stage (E14.5) and leads to the formation of the enamel knot and lateral protrusions called cervical loops (CL) at the lingual and labial parts. Finally, during the bell stage (E15.5 to E19.5), the inner enamel epithelium and the outer enamel epithelium are generated first, followed by the formation of 2 tooth specific cell populations: the enamel-producing ameloblasts and dentin-producing odontoblasts (Mina and Kollar, 1987; Lumsden, 1988; Ruch et al., 1995; Zeichner-David et al., 1995, 1997; Vaahtokari et al., 1996; Thesleff and Sharpe, 1997; Moradian-Oldak, 2001; Thesleff et al., 2001; Miletich and Sharpe, 2003). While odontoblasts are evenly distributed at the labial and lingual side of incisors in mice, the ameloblasts are localized only on the labial side. Consequently, enamel formation in mice incisors shows an asymmetric distribution (Tummers and Thesleff, 2003). This morphological cascade of events is achieved through the coordinated expression of key transcription factors such as Pax9, Pitx2, Runx2 and Msx1-2 (Thesleff, 2003) as well as signaling molecules such Bmp4, Fgf and Shh (Bei and Maas, 1998). In humans, mutations in some of these genes are associated to dentition problems such as oligodontia (Vastardis et al., 1996; Stockton et al., 2000). As previously mentioned, *Bcl11b* ± heterozygous C57BL/6 mice suffer from dentition-related pathologies (Inoue et al., 2017). Earlier studies reported similar observations and estimated that 25% of heterozygotes possess extended and fragile incisors (Golonzhka et al., 2009b). In line with these observations, several studies conducted detailed analyses into the role of Bcl11b in tooth formation in both embryos and adult mice. A critical role for Bcl11b in the formation of the labial and lingual epithelial stem cell populations and ameloblast differentiation has been established in embryonic development (Golonzhka et al., 2009b; Kyrylkova et al., 2012). The alteration of these tooth formation processes led to the development of incisors with reduced size and abnormal shape (Figure 1C). More specifically, during the bud stage, Bcl11b-deficient mice are characterized by a reduced proliferation of epithelial cells that remain poorly polarized (Figure 1B). Bmp4 signaling was also altered at this stage. While Bmp4 expression is unaffected in the dental mesenchyme of Bcl11b-deficient mice, its expression in the dental epithelium is not downregulated as observed in the control embryos (Figure 1B). At the cap stage, the hypoproliferation of epithelial cells was also a characteristic feature of mutant mice, probably due to the defective Fgf10 signaling in the underlying mesenchyme. At the bell stage, the main feature of mutant mice was the inversion in the expression pattern of Fgf family genes relative to the labial-lingual axis. This resulted in the inverted formation of a large lingual CL and reduced labial CL as well as the abnormal presence of ameloblast-like cells on the lingual aspect of the incisors (Figure 1B). In accordance to these observations, other factors (Sprouty, Shh) were also affected. Detailed analysis of the ameloblast lineage in Bcl11b-deficient mice showed a downregulated expression of crucial ameloblast markers such as amelogenin, enamelin and ameloblastin as well as Laminin 5-3a. The latter plays a critical role in the adhesion and integrity of the ameloblast cell layer and its expression is regulated by the Msx2 transcription factor (Aïoub et al., 2007). Results of ChIP experiments suggest that Msx2 is a direct transcriptional target of Bcl11b (Golonzhka et al., 2009b). Msx2-/- homozygous mice have reduced expression of Bmp4. Additionally, these mice exhibit dental and periodontal defects as well as altered amelogenesis (Aïoub et al., 2007; Babajko et al., 2015; Nakatomi et al., 2018). An important transcription factor in amelogenesis, Sp6, is also a direct transcriptional target of Bcl11b (Adiningrat et al., 2014; Smith et al., 2020). Homozygous mice (Sp6-/-) have severe defects in teeth, as well as defects in skin, hair follicles, and digit formation (Nakamura et al., 2008). They demonstrated that Sp6 inactivation leads to the dysregulation of Lef-1 and Shh expression and localization, defects in the differentiation of the dental epithelium and mesenchyme, and the formation of multiple premature enamel Knot-like structures. In adult mice, the observation that some of the *Bcl11b* ± heterozygous mice possess extended and fragile incisors suggests a dose-dependent effect of Bcl11b in incisor growth (Katsuragi et al., 2013). Indeed, defects in the maxillary incisors as well as shorter maxillary bone is observed in 3-week-old mice with a hypomorphic allele. The incisors were characterized by lower concentrations of calcium, phosphorus and ferrum. The authors suggest that these defects are related to a compromised proliferation/differentiation of the ameloblast lineage due to a strong expression of cell cycle inhibitors p27/p57.

In conclusion, Bcl11b plays a critical role at various stages of tooth formation during embryonic development. It would be reasonable to speculate that, in humans, the perturbation of Bcl11b expression levels and patterns could have an impact on the correct disposition of teeth during childhood.

### Bcl11b Is Required for Craniofacial Skeleton Formation

The sophisticated craniofacial formation requires the contribution of all three germ layers; the ectoderm, mesoderm and endoderm. BMP, TGF-ß, Wnt, FGF and hedgehog signaling pathways coordinate the morphogenetic processes involving several key transcription factors such as Hox, Msx1/2, Pitx1/2, Runx2, Sox9, and TCF/LEF. Among them, Runx2 is considered as the master transcription factor for the expression of major bone matrix protein genes. Normal skeletal development depends on its temporal- and spatial-specific expression. Early onset of Runx2 expression in the cranial mesenchyme in transgenic mice resulted in craniosynostosis, ectopic bone formation, and limb defects (Maeno et al., 2011), whereas the proliferation of osteoblast progenitors is impaired due to the reduced expression of the direct Runx2 targets Fgfr2 and Fgfr3 in Runx2−/− mice (Kawane et al., 2018). In heterozygous Runx2+/− mice, the process of posterior frontal and sagittal suture closure is completely interrupted. This interruption is linked to altered expression of genes implicated in hedgehog, Fgf, Wnt and Pthlh signaling pathways indicating that more than a half dosage of Runx2 is needed for correct suture closure (Qin et al., 2019). The role of Bcl11b has been recently examined in craniofacial skeleton formation. Analysis of Bcl11b expression patterns during embryonic development revealed its high-level of expression in craniofacial suture mesenchyme (Holmes et al., 2015; Kyrylkova et al., 2016). Skull formation during fetal development and its gradual expansion during the post-natal period are essential to achieve appropriate brain growth in humans. This process is regulated by the progressive ossification of the sutural mesenchyme. Indeed, premature ossification of sutures leads to craniosynostosis; a pathological condition affecting 1 in 2250 births. This condition is characterized by reduced skull size, increased intracranial pressure and craniofacial dysmorphologies (Rosenberg et al., 1997; Nie, 2005; Purushothaman et al., 2011). Genetic studies identified causal mutations in only 25% of cases; the most frequent being activating mutations in genes coding for FGF receptors (Cunningham et al., 2007; Purushothaman et al., 2011) as well as for other genes (Twist1, EFNB, etc.). As Bcl11b was shown to regulate FGF-dependent signaling pathways, analyses of Bcl11b-deficient mice as well as of neural crest-specific inactivation of the gene (*Bcl*11*b^*ncc*–/–^*) revealed its implication in craniofacial development and the maintenance of sutural patency (Holmes et al., 2015; Kyrylkova et al., 2016). *Bcl*11*b^*ncc*–/–^* mice survive for up to 3 weeks of age and exhibit severe midfacial hypoplasia with synostoses of facial skeleton. Germline deletion of Bcl11b shows additional abnormalities, as evidenced by the premature fusion of the temporal and coronal sutures (Figure 1D). Based on the expression level and domain of key osteogenic factors such as Runx2, Bone sialoprotein (Bsp) and the Fgfr2c receptor, the authors establish a working model placing Bcl11b upstream of these factors. Indeed, *Bcl*11*b^–/–^* facial skeleton exhibit increased expression of Runx2 in the osteogenic mesenchyme, and ectopic transcripts were detected in the coronal sutures of E16.5 embryos. Upregulated expression of Bsp, a marker of osteoblast maturation, was also evidenced in facial skeletons. As for Fgfr2c expression, the authors suggest that its ectopic expression in the facial and coronal sutures induces a ligand-independent activation of the receptor and participates in the premature ossification of the sutures leading to craniosynostosis. In humans, several cases of craniofacial abnormalities associated with Bcl11b mutations were reported. In a severe combined immunodeficiency (SCID) patient with multisystem defects including craniofacial abnormalities, a heterozygous missense mutation in the DNA-binding zinc-finger domain (ZFN2) of Bcl11b (p.N441K) was identified as conferring a dominant negative activity to the protein (Punwani et al., 2016; Table 1). Morpholino-mediated knockdown of the Bcl11b orthologous gene in zebrafish (Bcl11ba) as well as an overexpression of the mutated human Bcl11b gene reproduced similar defects in the immune system and the craniofacial structure. Furthermore, rescue experiments through the expression of the normal human Bcl11b gene reversed the phenotype of the zebrafish embryos, suggesting a causative genotype/phenotype link. A second case concerns a craniosynostosis, in which a *de novo* point mutation of the Bcl11b gene (p.R3S) was identified (Goos et al., 2019; Table 1). The generation of a mouse model with the identified mutation confirmed its causative role to some extent. Furthermore, the study uncovered the potential mechanism by which this mutation leads to the observed phenotype. Indeed, through the use of co-immunoprecipitation assays as well as biophysical methods (fluorescence anisotropy) and molecular modeling, the authors suggest that the *de novo* mutation impairs the interaction of Bcl11b with RBBP4 and 7, which are components of the NuRD and Prc2 complexes. The variability of the described phenotypes suggests a dose-dependent regulatory role of Bcl11b for the timely and coordinated differentiation of osteogenic precursors during the formation and growth of craniofacial bone structures.

**TABLE 1:** Bcl11b mutations in human diseases.

N°	Base change	Amino acid change	Mutation type	Mutation effect	Phenotype (associated diseases)	References
1	c.7C > A	p.R3S	MS	Reduced affinity between Bcl11b and RBBP4-MTA1 of NuRD complex	Coronal suture synostosis	Goos et al., 2019
2	c.242delG	p.C81Lfs*76	FS	Activation of the nonsense-mediated mRNA decay	Intellectual disability, speech delay, T cell and dental anomalies, dysmorphic facies	Lessel et al., 2018; Homma et al., 2019
3	c.1323T > G	p.N441K	MS	Mutation in C2H2 zinc finger, Dominant negative activity, alteration of BCL11B DNA binding activity	SCID, neurological, craniofacial and dermal abnormalities	Punwani et al., 2016
4	c.1333C > T	p.H445Y	MS	Mutation in C2H2 zinc finger, Dominant negative activity, alteration of BCL11B DNA binding activity	T-cell acute lymphoblastic leukemia (T-ALL)	Gutierrez et al., 2011
5	c.1340G > A	p.R447H	MS	Mutation in C2H2 zinc finger, Dominant negative activity, alteration of BCL11B DNA binding activity	T-cell acute lymphoblastic leukemia (T-ALL)	Gutierrez et al., 2011
6	c.1365_1367del3	p. Y455*	FS	Protein loss or, at least, loss of the last two C2H2 zinc fingers in the C-terminal domain.	Intellectual disability, speech delay, T cell anomalies, dysmorphic facies, allergy	Lessel et al., 2018
7	c.1495G > T	p. E499*	STOP	Protein loss or, at least, loss of the last two C2H2 zinc fingers in the C-terminal domain.	Intellectual disability, speech delay, T cell anomalies, dysmorphic facies, allergy	Lessel et al., 2018
8	c.1501dupC	p.T502Hfs*15	FS	Protein loss or, at least, loss of the last two C2H2 zinc fingers in the C-terminal domain.	Intellectual disability, speech delay, T cell anomalies	Lessel et al., 2018
9	c.1552delC	p.R518Afs*45	FS	Protein loss or, at least, loss of the last two C2H2 zinc fingers in the C-terminal domain.	Intellectual disability, speech delay, T cell and dental anomalies, dysmorphic facies, allergy	Lessel et al., 2018
10	c.1600delG	p.D534Tfs*29	FS	Protein loss or, at least, loss of the last two C2H2 zinc fingers in the C-terminal domain.	Intellectual disability, speech delay, T cell anomalies, dysmorphic facies	Lessel et al., 2018
11	c.1786G > A	p.G596S	MS	Mutation in Glycine-rich region	T-cell acute lymphoblastic leukemia (T-ALL)	Gutierrez et al., 2011
12	c.1944_1965del22	p.G649Afs*67	FS	Premature stop codon, truncated protein with 66 erroneous amino acids.	Intellectual disability, speech delay, T cell and dental anomalies, dysmorphic facies	Lessel et al., 2018 Prasad et al., 2020
13	c.2190_2200del11	p.T730Tfs*151	FS	Truncated protein lacking the C2H2 zinc fingers in the C-terminal domain	intellectual disability, speech delay, craniofacial abnormalities,	Qiao et al., 2019
14	c.2421C > G	p.N807K	MS	Mutation in C2H2 zinc finger, Dominant negative activity, alteration of BCL11B DNA binding activity	Intellectual disability, speech delay, T cell and dental anomalies, dysmorphic facies, allergy	Lessel et al., 2018 Prasad et al., 2020
15	c.2449_2456dup8	p.G820Afs*27	FS	Protein loss or, at least, loss of the last two C2H2 zinc fingers in the C-terminal domain.	Intellectual disability, speech delay, T cell and dental anomalies, dysmorphic facies	Lessel et al., 2018
16	c.2539G > C	p.G847R	MS	Mutation in C2H2 zinc finger, Dominant negative activity, alteration of BCL11B DNA binding activity	T-cell acute lymphoblastic leukemia (T-ALL)	Gutierrez et al., 2011
17	c.2671delG	p.A891Pfs*106	FS	Protein extension with erroneous 103 amino acids, probably triggering non-stop mRNA decay	Intellectual disability, speech delay, T cell anomalies, dysmorphic facies, allergy	Lessel et al., 2018

*MS: missense; FS: Frameshift.*

### Bcl11b Mutations and Genetic Polymorphisms

Bcl11b mutations in T-ALL has been reported (Gutierrez et al., 2011; Table 1), they include mutations affecting zinc finger domains (p.H445Y, p.R447H, p.G847R) or Glycine-rich region (p.G586S). These include mutations affecting zinc finger domains (p.H445Y, p.R447H, p.G847R) or glycine-rich regions (p.G586S). In a study concerning 13 cases of patients with developmental delay, moderate intellectual disability and mild facial dysmorphisms accompanied by impaired T cell development and a severe reduction in peripheral ILC2 cells, 10 monoallelic mutations at the *Bcl11b* locus were found (Lessel et al., 2018; Table 1). Eight of ten variants are frameshift mutations. Among them, p.C81Lfs^∗^76 and pA891Pfs^∗^106 are predicted to result in haploinsufficiency. The remaining six frameshift mutations and one codon stop mutation, p.T502Hfs^∗^15, p.R518Afs^∗^45, p.D534Tfs^∗^29, p.G649Afs^∗^67, p.G820Afs^∗^27, p.Y455^∗^ and p.499^∗^, are predicted to result in a truncated protein lacking C-terminal zinc finger domains. One missense mutation p.N807K affects the alpha-helix containing the DNA recognition site within the zinc finger domain. Detailed study of the p.G820Afs^∗^27 mutation showed its inability to rescue the cell proliferation defect in Bcl11b KO hippocampal slice cultures. Three of these mutations (p.C81L76^∗^fs, p.G649Afs^∗^67 and p.N807K) were also reported in other patients (Homma et al., 2019; Prasad et al., 2020). Finally, a *de novo* heterozygous frameshift mutation (p.T730Tfs^∗^151) was identified in a 5-year-old girl with craniofacial abnormalities consisting of micrognathia, hypertelorism and short palpebral fissures. To reiterate, this mutation is predicted to produce a truncated protein lacking C-terminal zinc finger domains (Qiao et al., 2019). The aforementioned mutations are presented in Table 1.

**FIGURE 2:**
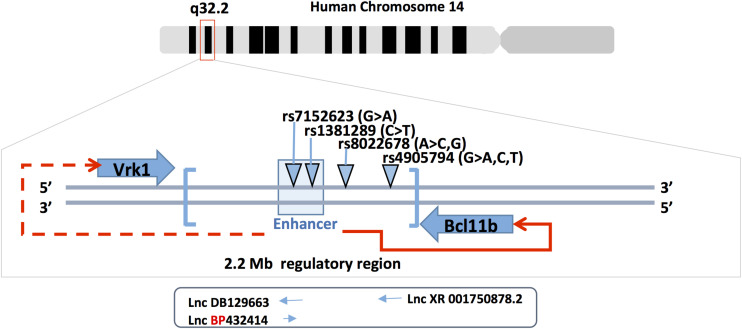
Bcl11b gene locus and SNPs. Bcl11b is located on human chromosome 14q^32^ (upper panel). The middle panel shows the position of Bcl11b and Vrk1 genes, the 3’ Bcl11b gene desert (in brackets), the enhancer region at 3’ of Bcl11b (light-blue box) and the four SNPs involved in cardiovascular phenotype. The enhancer is functionally involved in the regulation of Bcl11b gene expression (red arrow) but less likely in Vrk1 gene expression (doted red arrow). Three long non-coding RNAs are schematically represented under the corresponding SNPs (rs7152623 and rs4905794).

In addition to the mutations in the coding sequence of Bcl11b, several studies reported single nucleotide polymorphisms (SNPs) in the 3’gene desert of Bcl11b that seems to modulate its expression. The expression level of Bcl11b seems to be a crucial parameter for outcomes in terms of cellular response, gene activation/repression and tissue homeostasis. The described SNPs were found in the 3’ region of Bcl11b that harbors gene regulatory sequences as well as long non-coding RNA (Lnc-RNAs) sequences. An initial human GWAS-based meta-analysis that included more than 20000 participants and 2 replication cohorts (over 5000 participants) showed that common genetic variations in the 3’ Bcl11b gene desert (rs1381289 and rs7152623) are associated with a higher susceptibility for aortic stiffness and increased risk for cardiovascular disease. This conclusion was based on the measure of carotid-femoral pulse wave velocity, which is an aortic stiffness parameter (Figure 2) (Mitchell et al., 2012). In addition, the study showed the presence of RNA transcripts for the 2 Lnc-RNAs (DB129663 and BP432414), Bcl11b and the VRK1 gene (another gene present near the SNP block) in aortic smooth muscle cells and human aortic rings. The presence of these transcripts in the tissue of interest (aortic smooth muscle cells) suggests that the SNPs could affect regulatory mechanisms that control Bcl11b and, less likely, VRK1 expression through modification of the Lnc-RNAs properties or enhancer activity. Recently, a study investigating the role of Bcl11b in aortic stiffness confirmed the correlation between specific SNPs and Bcl11b expression levels (Maskari et al., 2018). They suggest that the impact of Bcl11b on arterial stiffness could be related to its presence in infiltrating cells and, more specifically, type-2 innate lymphoid cells. ILC2 cells are resident lymphoid cells that play a protective role in atherosclerosis for which type 2 cytokines IL-5 and IL-13 are required (Newland et al., 2017). The authors further discuss the possibility that the VRK1 gene and the Lnc-RNAs present in the SNP locus could play a role in arterial stiffness. However, smooth muscle cell specific modulation of Bcl11b expression is not excluded as playing a role in arterial stiffness. A preprinted study in BioRxiv (Valisno et al., 2017) that requires peer- review validation suggests such a possibility.

Two additional GWAS studies reported SNPs at the 3’ region of the Bcl11b gene that achieved genome-wide significance (Figure 2). The first study identified the rs4905794 to be significantly associated with nocturnal blood pressure dipping in hypertensive patients (Rimpelä et al., 2018). The analysis was performed on a small cohort (204 patients; GENRES cohort). However, the two replication cohorts consisting of approximately 180 patients (DYNAMIC and DILGOM cohorts) failed to reach statistical significance. The second study investigated the genetic factors that modulate the effect of sodium on blood pressure in Japanese populations (Hachiya et al., 2018). A total of nearly 8700 patients in two independent cohorts were analyzed. The study resulted in the identification of a novel SNP in the 3’ locus of Bcl11b (rs8022678). The authors classified the cohort for this SNP into sodium-sensitive A and sodium-insensitive A non-carriers. Furthermore, they subdivided the patients into low, medium and high sodium consumption groups and, based on their systolic and diastolic mean blood pressure values, suggested that the A-carrier population would benefit from lower salt intake. While no clear mechanism was proposed, the authors suggest taking into account the discovered polymorphism in the context of personalized medicine for hypertensive patients. These various studies point to a potential role of Bcl11b in the cardiovascular system either through direct impact on vascular cells or indirect immune cell-mediated mechanisms.

Expression of Bcl11b as well as the surrounding genes and non-coding RNAs could be influenced by the SNPs located in the 3’ gene desert of Bcl11b. Such a possibility is supported by the results obtained in T cells. The expression of Bcl11b was shown to be largely dependent on a far downstream enhancer (Li et al., 2013). More recently, the Bcl11b enhancer was shown to relocate from the lamina to the nuclear interior in developing T cells (Isoda et al., 2017). The transcription of a non-coding RNA named ThymoD promoted demethylation at CTCF bound sites and activated cohesin-dependent looping to reposition the Bcl11b enhancer from the lamina to the nuclear interior as well as to juxtapose the Bcl11b enhancer and promoter into a single-loop domain. These large-scale changes in nuclear architecture are in agreement with the hypothesis of the two parallel cis and trans rate-limiting steps for the control of Bcl11b gene transcription (Ng et al., 2018). The cis rate-limiting step consists of a switch of the Bcl11b locus from an inactive to active epigenetic state. The trans rate-limiting step concerns the Notch-dependent activation of trans factors, which modulate the transcription of the cis-activated Bcl11b locus. This model is in accordance with the fact that Bcl11b expression varies not only between cells but also between Bcl11b alleles within the same cell (Ng et al., 2018). It is plausible that minimal variations in Bcl11b regulatory sequences such as SNPs could alter these complex regulatory mechanisms, affect Bcl11b expression (increase/decrease) and thus induce physiological changes in arterial stiffness or blood pressure.

## Concluding Remarks and Perspectives

The multisystem impact of Bcl11b mutations and SNPs in humans as well as the observed phenotypes in animal models suggests that Bcl11b, through its multiple partners, DNA interaction domains and diversity of target genes, regulates key processes in multiple tissues during embryonic development as well as in adulthood. Despite the apparent variability in terms of physiological outcomes in each of these tissues, common cellular and molecular features can be defined for Bcl11b. First, Bcl11b seems to control the balance between cell proliferation and differentiation during organ formation and repair and, more specifically, in the context of stem cell self-renewal and fate determination. This is achieved partly through the regulation of cell cycle genes (p21, p57kip2…) as well as components of critical signaling pathways (Bmp4, Fgf, Notch, Wnt…). Second, Bcl11b seems to play a protective role against cell death in healthy tissue but can also enhance this process under pathological conditions as observed for CTCL tumor cells. It would be interesting to orient future research on coupling Bcl11b and its related epigenetic actors (NuRD, SWI/SNF, pTEFb complexes) to the physiological response observed under various situations (health, disease, aging, etc.). Future studies should aim to identify common and tissue-specific Bcl11b target genes and mechanism of action in various tissues through the combination of different state-of-art approaches such as ChIP-sequencing, ATAC, and RNA-seq at the level of tissue or single cells. This was successfully achieved in T cells and ILC2 cells and should serve as a model for other tissues. The main challenge for other solid tissues might be the isolation process, which requires mechanical and enzymatic treatments. These manipulations can lead to changes in chromatin state. Given that Bcl11b was shown to cooperatively bind to DNA and interact with key transcription factors for T-cell subset identity (Foxp3, Gata-3…), it will be interesting to determine if this is also the case in other cells such as keratinocytes, adipocytes and osteogenic progenitors. Finally, its established role in regulating expression (repression) of different viral genes as well as its key role in the immune response motivates the search for a potential role of Bcl11b for viral, bacterial and parasite infections, with the objective of modulating its expression through drug screening strategies.

## Author Contributions

M-TD, ZL, and AP performed literature search, drafted the manuscript and generated the figures. PB and OA performed critical reading of the manuscript and provided insights for the figures. All authors contributed to the article and approved the submitted version.

## Conflict of Interest

The authors declare that the research was conducted in the absence of any commercial or financial relationships that could be construed as a potential conflict of interest.
